# Accuracy of acetabular cup placement during anterolateral supine total hip arthroplasty using intraoperative fluoroscopy: a retrospective study

**DOI:** 10.1186/s13018-022-03422-9

**Published:** 2022-12-05

**Authors:** Eiji Cho, Takashi Hisatome, Shuhei Oda, Hirohisa Fujimaki, Kazuyoshi Nakanishi

**Affiliations:** 1 Department of Orthopaedic Surgery, Nihon University Hospital, Tokyo, Japan; 2 Department of Orthopaedic Surgery, Nihon University Itabashi Hospital, Tokyo, Japan

**Keywords:** Intraoperative fluoroscopy, Total hip arthroplasty, Anterolateral supine approach, Component position, Accuracy

## Abstract

**Background:**

In our institution, total hip arthroplasty (THA) is performed using the anterolateral supine (ALS) approach with intraoperative fluoroscopy. This study aimed to investigate and review the accuracy of acetabular cup placement in ALS-THA using intraoperative fluoroscopy.

**Methods:**

A total of 142 patients with 154 joints (mean age 64.3 years, 30 males and 112 females) underwent ALS-THA with intraoperative fluoroscopy at the same institution. The target angle of the cup position was set at 40° for radiographic inclination (RI) and 5°–25° for radiographic anteversion (RA) based on the functional pelvic plane according to the pelvic motion during individual postural changes. The cup position angle was measured using postoperative computed tomography, and the error in the target angle was investigated.

**Results:**

The target angle of RI was 40°, and the postoperative RI was 39.3° ± 4.3°. The target angle of the RA was 17° ± 2.6°, and the postoperative RA was 20.6° ± 3.7°. The absolute values of the error from the target angle were 3.6° ± 2.5° for RI and 4.2° ± 3.3° for RA.

For RI and RA, 67.5% (104/154 joints) were within ± 5° of the target and 96.1% (148/154 joints) were within ± 10°.

**Conclusions:**

The accuracy of cup positioning in ALS-THA using intraoperative fluoroscopy was good and appeared comparable to that of various navigation systems.

## Background

In total hip arthroplasty (THA), accurate cup placement is important for good long-term results and avoidance of complications. Cup malposition leads to postoperative dislocation, polyethylene wear, and decreased range of motion [1].

Cup placement using free-hand techniques is performed using bony landmarks, such as the superior anterior iliac spine and pubic symphysis; however, it is difficult for even high-volume surgeons to perform accurate placement in all cases [2]. In recent years, intraoperative tools, such as computed tomography (CT)-based navigation systems and robot-assisted surgery, have been used for accurate cup placement, and both have reported precise cup placement accuracy [3–5]. However, these CT-based navigation systems and robot-assisted surgeries are not common because of the limited number of institutions where they can be used or their cost.

The angle of cup placement is also affected by whether it is performed in the lateral or supine positions. Many reports indicate that supine positions, such as the direct anterior approach (DAA) and anterolateral supine (ALS) position, are more accurate for cup placement and have a lower risk of dislocation than the lateral decubitus position [6, 7].

Cup placement is considered more accurate in the supine position because the pelvis is stabilized intraoperatively; however, it is not accurate in all institutions and depends on the surgeon's skill [8]. One way to avoid these implant malpositions and complications is through intraoperative fluoroscopy. Fluoroscopy is a must-have device in every institution and is especially easy to use in the supine position. Intraoperative fluoroscopy can reduce the frequency of implant malpositioning because it can confirm the anteversion and inclination of the cup. On the femoral side, femoral offset, femoral head center, and leg length reconstructions are also possible.

This study aimed to investigate the accuracy of cup placement in ALS-THA using intraoperative fluoroscopy. The error between the target cup angle and the postoperative cup angle inserted based on intraoperative fluoroscopy was measured, and its accuracy was examined.

## Methods

### Patients

The study was approved by a single institution. This was a retrospective review of 154 hips in 142 patients (30 hips in 30 males and 124 hips in 112 females) who underwent primary THA via the ALS approach from April 2021 to March 2022. The mean age was 64.3 years, and the mean postoperative follow-up was 1.5 years. The average height, weight, and BMI were 156.7 cm, 57.9 kg, and 23.4 23.4 kg/m^2^, respectively. The preoperative diagnoses were osteoarthritis in 129 hips and avascular necrosis of the femoral head in 25 hips (Table 1).

**Table 1:** Patient demographics

	*n* = 154
Age: years ± SD	64.3 ± 9.3
Sex: male/female	30 patients 30 hips/112 patients 124 hips
Height: cm ± SD	156.7 ± 0.1
Weight: kg ± SD	57.9 ± 12.3
Body mass index: kg/m^2^ ± SD	23.4 ± 3.7
Treated side: right/left	48/106
Diagnosis: OA/ONFH	OA 129/ONFH 25
Crowe: G1/2/3/4	121/25/8/0

*SD* standard deviation, *OA* osteoarthritis, *ONFH* osteonecrosis of the femoral head

### Surgical procedures

**Fig. 1:**
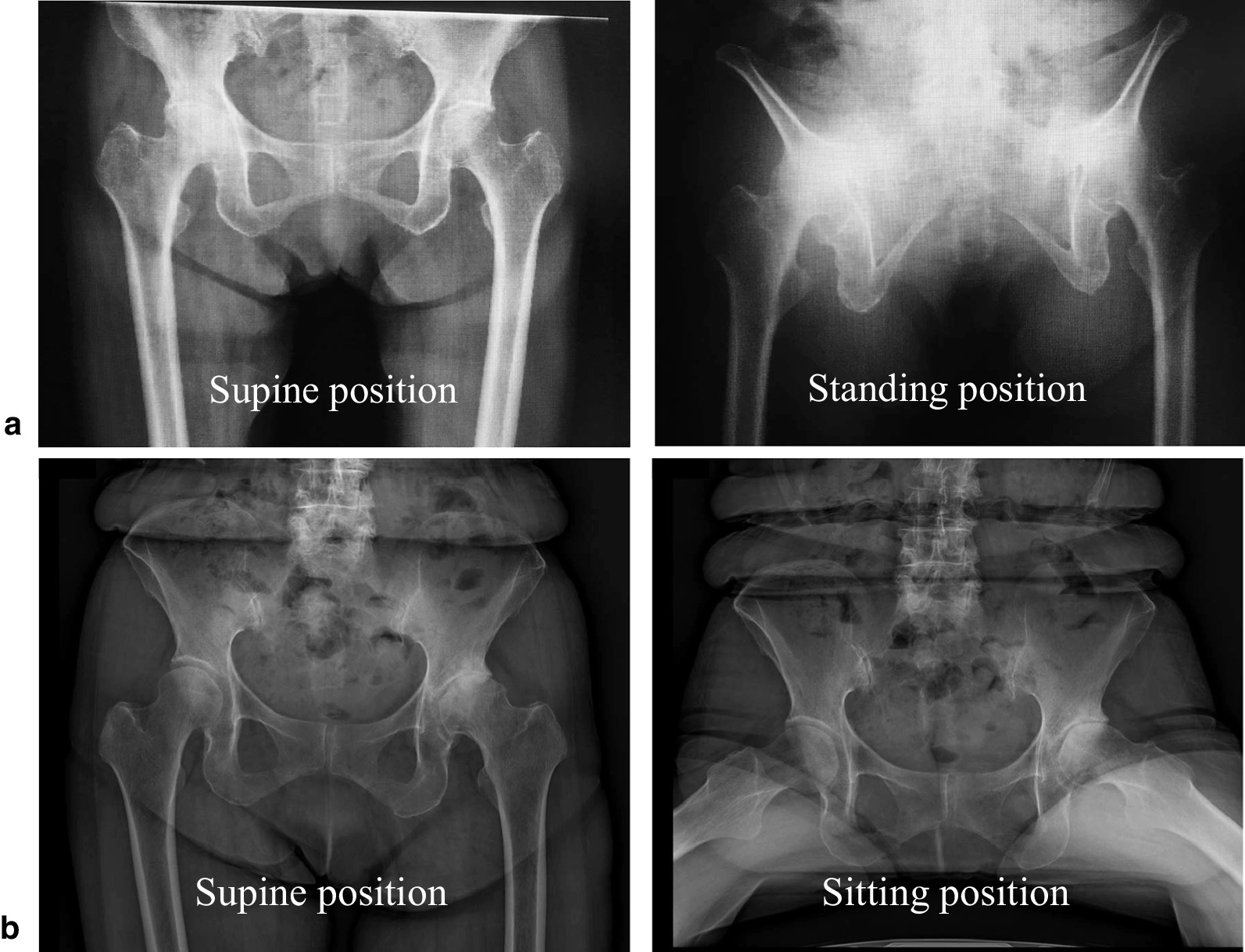
Pelvic postural change in the supine, standing, and sitting positions. **a** When the posterior tilt during supine to standing position is large, the target radiographic anteversion (RA) is 5° or 10°. **b** When the posterior tilt during supine to the sitting position is small, the target RA is 20° or 25°

All surgeries were performed by four surgeons on a standard operating table using the same technique. Surgery was performed under general anesthesia. Preoperative AP bilateral hip radiographs in the supine, standing, and sitting positions were obtained in all cases to evaluate pelvic motion during postural changes. The target angle of radiographic anteversion (RA) was set to 15°. The target angle of the RA was set at 20° or 25° for cases with stiff pelvic motion during a postural change from a standing to a seated position. The RA target angle was set at 5° or 10°°for cases with a large posterior pelvic tilt in the supine to standing position (Fig. 1). The target angle of the radiographic inclination (RI) was 40° in all cases. No 2D or 3D template software was used as a preoperative template. The approximate cup size and femoral anteversion were measured using CT as an intraoperative reference.

**Fig. 2:**
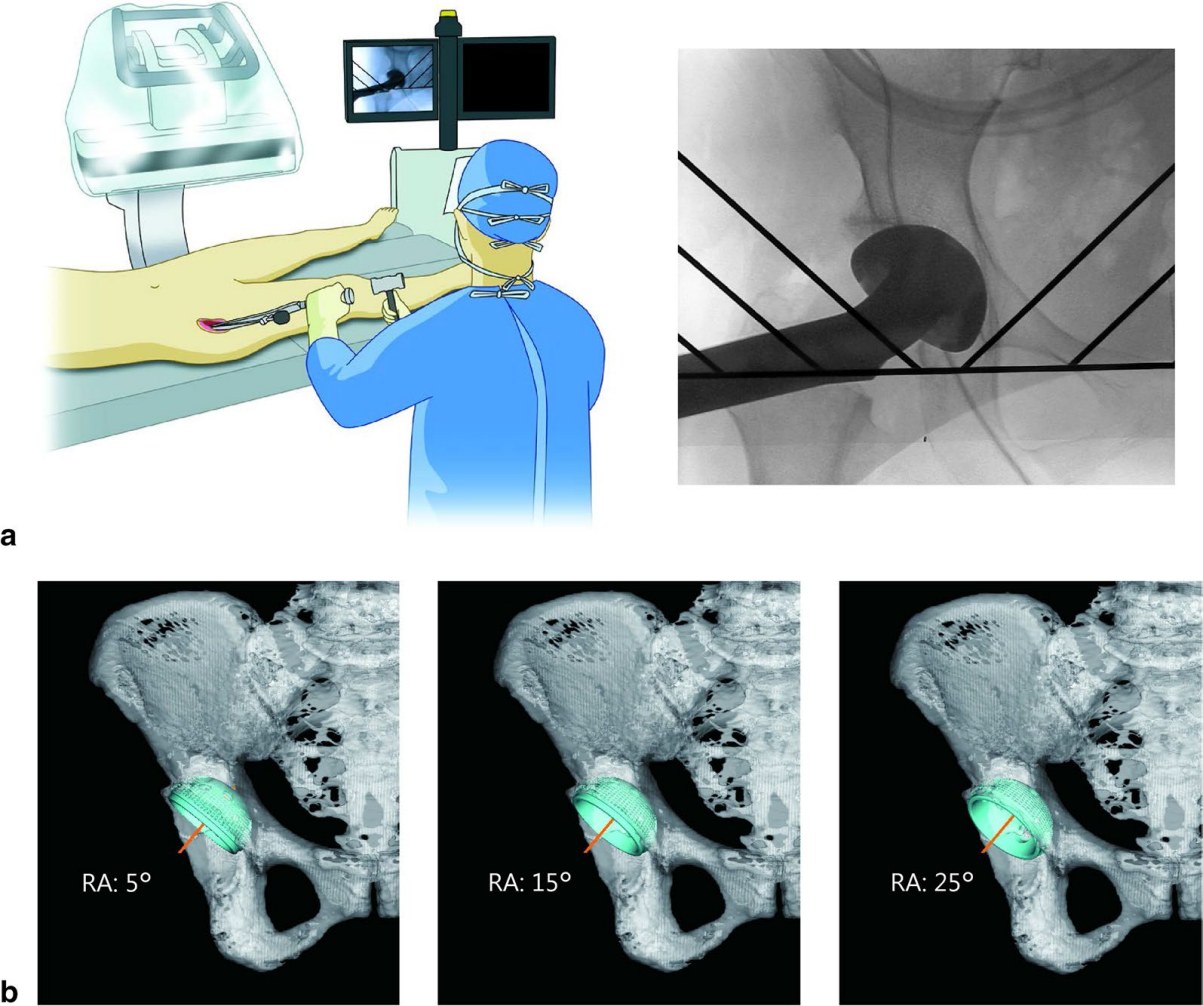
Cup placement using intraoperative fluoroscopy. **a** The surgeon estimated the target angle during cup seating using intraoperative fluoroscopy. **b** Reference diagram as an indicator of cup radiographic anteversion (RA) (5°, 15°, 25°)

After general anesthesia, the patient's bilateral hip joints were visualized fluoroscopically. The same fluoroscopy model (12-inch, General Electric OEC 9900, USA) was used in all the cases. The angle of incidence of fluoroscopy was adjusted so that the shape of the pelvic cavity and obturator foramen were similar on both sides, referring to the AP view of both hip joints in the supine position performed preoperatively. The acetabulum was reamed on the ilioischial line using fluoroscopy. The cementless cup was placed at the target angle for each fluoroscopy case without using a mechanical alignment guide. When seating the cups, all retractors were removed to prevent movement in the pelvis. Cup alignment was also checked fluoroscopically during cup placement, after screw insertion, and after liner placement, as appropriate. The cup was seated with visual confirmation using a pre-prepared schema to indicate cup anteversion and inclination (Fig. 2).

### Evaluation

One week postoperatively, CT was performed in the supine position. RI and RA were measured using 3D template software (Zed Hip, LEXI, Tokyo, Japan). Measurements were performed by two observers, and the average value was used. The functional pelvic plane (FPP) was defined as the reference plane; the same size cup was used to overlap the seated cup completely, and its angle was measured. To assess the accuracy of cup placement, we calculated the percentage of cup position in Lewinnek's safe zone [9]. The error between the measured RI/RA and the preoperative target angle was calculated. Errors were evaluated as average and absolute values. In addition, the percentage of absolute values of errors within 5° and 10° was calculated.

## Results

The average preoperative target RI was 40°, and the RA was 17° ± 2.6°. The average postoperative RI was 39.3° ± 4.3°, and the RA was 20.6° ± 3.7°. A total of 98.1% (151/154) of the joints were within Lewinnek’s safe zone. The average error from the target was − 0.7° ± 4.3° for RI and 3.5° ± 4.0° for RA. The absolute error from the target was 3.6° ± 2.5° for RI and 4.2° ± 3.3° for RA (Table 2). For RI and RA, 67.5% (104/154) of the joints were within ± 5° of the target and 96.1% (148/154) were within ± 10° (Table 3). For RI, 81.2% (125/154) of the joints were within ± 5° of the target and 100% (154/154) were within ± 10°. For RA, 80.5% (124/154 joints) and 97.4% (150/154 joints) of the participants had errors within ± 5° and ± 10° of the target, respectively.

**Table 2:** Average values and absolute values of differences in postoperative angle from the target angle

	RI (°)	RA (°)
Target angle	40	17 ± 2.6
Postoperative angle	39.3 ± 4.3	20.6 ± 3.7
Average values of differences	− 0.7 ± 4.3	3.5 ± 4.0
Absolute values of differences	3.6 ± 2.5	4.2 ± 3.3

**Table 3:** Accuracy in cup placement for the target angles

	within ± 5°	within ± 10°
RI and RA	67.5% (104/154 hips)	96.1% (148/154 hips)
RI	81.2% (125/154 hips)	100% (154/154 hips)
RA	80.5% (124/154 hips)	97.4% (150/154 hips)

*RI* radiographic inclination, *RA* radiographic anteversion

## Discussion

Intraoperative tools, such as CT-based navigation systems and robot-assisted surgery, are used for accurate cup placement, and precise cup placement accuracy has been reported. However, their use requires registration to obtain accurate location information, and individual preoperative planning is time-consuming and expensive. In recent years, simpler portable navigation systems have been developed, and their accuracy has been reported.

The accuracy of cup placement by various portable navigation systems in the supine position is 2.6°–3.7° for RI and 2.8°–3.8° for RA [10–12]. Okamoto et al. reported that in DAA-THA, the cup placement rate within ± 5° was 56.6% in the alignment guide group and 72.2% in the portable navigation (Hip Align^®^) group [13]. The accuracy of cup placement using various portable navigation systems in the lateral decubitus position is 2.5°–4.6° for RI and 2.3°–6.5° for RA [4, 14].

Supine approaches, such as DAA and ALS, have the advantage of easy fluoroscopy access. Fluoroscopy is available at all institutions and is easy to perform without additional costs. In this study using intraoperative fluoroscopy, Lewinnek's safe zone achievement rate was 98.1% (151/154 joints), and the accuracy of individual RI/RA was within ± 5° at > 80%. This result is comparable to those of studies using portable navigation, indicating the usefulness of intraoperative fluoroscopy. There have been reports of the usefulness of using fluoroscopy intraoperatively, as in this study, and it is said to contribute to improving the accuracy of cup placement [15]. On the other hand, the use of fluoroscopy does expose the patient and surgical staff to radiation. Fluoroscopy is often used in orthopedic surgery, even for common fracture surgery, and radiation exposure is an unavoidable problem for orthopedic surgeons and staff. Jinnai et al. reported significantly shorter fluoroscopy time and lower radiation exposure level for DAA-THA compared to proximal femur fractures [16]. In a recent systematic review, it was also reported that radiation exposure level during anterior THA is low and does not affect the surgeon or patient [17]. Therefore, we consider THA using fluoroscopy to be a safe procedure.

We targeted RI 40° and RA 15°, following Lewinnek's safe zone concept. However, in recent years, there have been many reports of dislocations, even in cases within the safe zone [18]. Concepts such as “functional safe zones,” in which cups are seated considering individual spinopelvic mobility, have been proposed [19]. Spinal parameters and pelvic motion have large personal deviations, and there are still many unknown and no established goals. However, we believe it is important to reduce outliers from the target range, as systematic reviews have shown that the risk of postoperative dislocation increases with deviation from the target range [20]. In our institution, preoperative AP radiographs of the bilateral hips in the supine, standing, and sitting positions were obtained to assess pelvic motion during postural changes. In general, the posterior pelvic tilt from supine to standing position was 5° and from standing to sitting was 20° [21]. However, some cases of pelvic positional changes differ between these motions. A large posterior pelvic tilt in the supine to standing position increased the risk of anterior dislocation in the hip extension position. A small posterior pelvic tilt from the supine to sitting position is associated with a high risk of posterior dislocation due to anterior impingement in the hip flexion position. The target RA value was adjusted to account for these pelvic motions in postural change, and there were no cases of dislocation or other complications in the short term, averaging 1.5 years postoperatively. However, further studies are required to determine these target angles.

Our study had several limitations. First, this was a retrospective study, not a randomized study. Long-term dislocation rates and clinical outcomes have not yet been evaluated. The angle of the cup position should be investigated further, as it affects clinical outcomes and complications. In addition, the surgeons were not identical, and each of the four surgeons had different years of experience. Differences in cup placement accuracy with years of experience have also been reported but were not examined among surgeons in this study [22]. However, we believe inter-operator error is minimized because all four fixed surgeons participated in the surgery in all cases and evaluated the angle of the cup position. Most patients in this study had few obesity and Crowe types I or II, with only mild deformities. There are reports that obese patients and patients with severe deformities are less accurate in navigation, so it is possible that the results would have been different if more obese or deformed patients had been studied [3]. Finally, as described above, there is the issue of radiation exposure. Our technique uses fluoroscopy, as appropriate, during cup placement. The more time fluoroscopy is used, the more exposed physicians, nurses, and others are. Although not done in this study, we believe that it is necessary to evaluate the time spent using fluoroscopy and make efforts to reduce the use of fluoroscopy.

## Conclusion

The accuracy of cup positioning in ALS-THA using intraoperative fluoroscopy was investigated. The accuracy of the cup position with respect to the target angle was good and appeared comparable to those of various navigation systems. Intraoperative fluoroscopy was considered useful for accurate cup placement in ALS-THA.

## Data Availability

The data presented in this study are available upon request from the corresponding author. The data are not publicly available due to regulations of the local institutional ethics board.

## Abbreviations

THA: Total hip arthroplasty
ALS: Anterolateral supine
RI: Radiographic inclination
RA: Radiographic anteversion
DAA: Direct anterior approach
